# Hepatotoxicity with cholestatic pattern secondary to enoxaparin treatment

**DOI:** 10.1515/almed-2021-0048

**Published:** 2021-11-03

**Authors:** Guillermo Velasco de Cos, Isabel Sánchez-Molina Acosta, Marta Iturralde Ros, Ainhoa Maiztegi Azpitarte, Ana Moyano Martinez

**Affiliations:** Hospital Universitario Marqués de Valdecilla, Santander, Cantabria, Spain; Hospital Comarcal de Laredo, Laredo, Cantabria, Spain

**Keywords:** cholestasis, enoxaparin, hepatitis, toxicity, transaminases

## Abstract

**Objectives:**

Treatment with low-molecular-weight heparins is very common in clinical practice. Exceptionally, some patients develop hepatitis within a few days of starting treatment, and rapid discontinuation of the drug is decisive to avoid chronification.

**Case presentation:**

These episodes usually present themselves only with elevated transaminases. We describe the case of a patient who presented a cholestatic pattern, which is very uncommon with this type of drug.

**Conclusions:**

Hepatitis secondary to heparin therapy is an underdiagnosed entity. The laboratory plays a fundamental role in its diagnosis, given that it initially presents itself without a clear clinical profile.

## Introduction

Anticoagulant therapy with unfractionated heparin (UFH) has been used since 1939. Its most prominent adverse effects are the risk of bleeding, thrombosis associated with thrombocytopenia, and antibody-mediated thrombocytopenia. At the end of the 20th century, low-molecular-weight heparin (LMWH) was marketed, which has fewer adverse effects than UFHs. In addition, their dosage adjustment is simpler since they do not require periodic analytical controls [1].

Among the adverse effects of both LMWH and UFH, the appearance of acute hepatitis stands out. The number of cases described is very limited, so the prevalence is not clear. According to the latest definitions of heparin-induced hepatotoxicity, this occurs when there is an increase of more than three times the upper limit of the reference interval in the value of transaminases, which cannot be justified by other causes [2]. Hepatotoxicity is estimated to occur in 5% of patients treated with UFH and 5–10% of patients treated with LMWH [3]. Within the latter, the presentation is variable depending on the drug. Tinzaparin was withdrawn from the market in 2011 for presenting this adverse effect in 13.3% of patients. On the other hand, of the two most commonly used currently, deltaparin is the one associated with the highest incidence of hepatitis, with cases reported with enoxaparin being rare [4].

LMWH hepatotoxicity can be divided into two patterns, cholestatic and hepatocellular, the latter being more frequent in most patients. The hepatocellular pattern shows increased alanine aminotransferase (ALT) and aspartate aminotransferase (AST), with hardly any elevation of alkaline phosphatase (ALP) and gamma-glutamyl transferase (GGT). On the other hand, the cholestatic pattern is defined by the elevation of ALP twice above the upper level of the reference range and an ALT/ALP ratio <2. This pattern occurs in about 30% of drug-induced hepatitis, with longer survival compared to the hepatocellular pattern. In both patterns, liver enzymes normalize after drug withdrawal. However, in the cholestatic pattern, this decline is slower and there is a greater likelihood of chronification [5].

Early diagnosis is a key. High bilirubin, ALP, and prothrombin time values are associated with increased severity and risk of chronification.

## Case presentation

We describe the case of an 83-year-old male patient diagnosed with Parkinson’s disease and type 2 diabetes mellitus, under treatment with ropinirole, carbidopa/levodopa, and metformin/sitagliptin. The patient did not consume alcoholic beverages, herbal products, tobacco, and had no other toxic habits.

Currently, he is being followed up by the Hematology Service for nondeficiency arregenerative anemia in the context of a chronic lymphoproliferative syndrome in treatment with rituximab and with unfilial gastrointestinal bleeding. The patient underwent an exploratory laparotomy to determine the origin of the bleeding, after which paracetamol and enoxaparin were prescribed for seven days.

The patient came for consultation one week after the operation, where an acute alteration in liver function tests was observed: AST: 128 U/L (14–35 U/L), ALT: 177 U/L (10–49 U/L), GGT: 650 U/L (<73 U/L), ALP: 428 U/L (46–116 U/L), TSH: 1.57 mU/L (0.55–4.78), with normal total bilirubin, and without associated symptoms.

Prior to surgery, and after the administration of two doses of rituximab, he had an unaltered liver profile, with negative serologies and a hemogram with hemoglobin: 8.1 g/dL (12–15.5 g/dL) and a hematocrit: 23.5% (34–46%).

In view of these new analytical findings, it was decided to perform an abdominal ultrasound, in addition to requesting serologies for hepatitis A, B, C, E, and cytomegalovirus. The ultrasound showed a liver with smooth contours, with discretely granular parenchyma and lithiasic residues inside the gallbladder. The negative results of the serologies for the different hepatitis viruses ruled out the viral origin of the infection. The serologies were repeated and again the same results were obtained. Autoimmune etiology was ruled out due to negative results.

After seven days a new analytical control was performed, showing normalization of transaminases AST: 13 U/L, ALT: 54 U/L, with persistent elevation of GGT: 317 U/L and ALP: 218 U/L. Total bilirubin remained in normal values. Two months after the onset of the episode, the patient continues with elevated GGT and ALP values: 132 U/L and 110 U/L, respectively. The evolution of the liver function parameters analyzed is shown in Table 1.

**Table 1:** Liver function evolution.

	Baseline state	Day 1	Day 7	2nd month	3rd month	7th month
GGT (<73 U/L)	54	650	317	132	105	63
ALP (46–116 U/L)	87	428	218	110	104	95
AST (14–35 U/L)	13	128	13	17	20	18
ALT (10–49 U/L)	12	177	54	53	16	12
Total bilirubin (0.2–1.1 mg/dL)	0.6	0.5	0.4	0.4	0.5	0.7

GGT, gamma-glutamyl transferase; ALP, alkaline phosphatase; AST, aspartate amino transferase; ALT, alanine amino transferase.

## Discussion

The mechanism by which heparin-induced hepatotoxicity occurs is unclear. In recent years, studies have been published that could relate the damage to the increase in HMGB1 protein, which is released from the cells after necrosis processes and acts as a signal of the innate immune response. It has been observed that, after the second day of heparin administration, this protein increases and it is postulated that it could induce cellular toxicity indirectly [4]. There are also publications advocating direct cytotoxicity, since after enoxaparin administration an increase in the serum level of miR-122, a biliary cell-specific intracellular biomarker, is observed [6].

Another possible mechanism is an alteration in hepatocyte membrane permeability. However, the latter mechanism is rare in drugs that are metabolized by desulfation, as is the case with LMWH [5].

To reach a final diagnosis, it is necessary to rule out other causes of liver damage, such as viral infections, toxic habits of the patient, and autoimmunity. In our case, the patient did not have toxic habits and the viral and autoimmune etiology were ruled out due to negative results.

Hepatotoxicity appears to be related to the sex and age of the patient, as well as to the dose of drug administered. Older males seem to be more predisposed to this complication [7]. However, the studies available to date are not very representative as they are based on very small sample sizes. The analytical alteration of the hepatic profile, in most cases, begins five days after the start of treatment [8], reaching maximum levels at approximately seven days and recovering normal values two weeks after discontinuation of treatment [9]. In our case, the onset of the alteration in the liver profile and the peak in the values of the parameters analyzed coincide with those described in the literature. However, recovery to baseline was much more prolonged, which would fit with the tendency to chronicity described for cases with a cholestatic pattern.

It is estimated that between 5 and 13% of patients with cholestatic patterns eventually develop chronic liver disease, and between 5 and 14% die or require long-term transplantation. The best predictors of mortality are transaminase and bilirubin levels. In this pattern, bilirubin values are variable, although they appear elevated more frequently than in the hepatocellular pattern, thus being associated with greater severity of the process [5]. The normal bilirubin levels and the discrete elevation of transaminases that the patient presented indicate a lesser severity of the alteration.

Clinically, drug-induced cholestasis presents with jaundice, pruritus, nausea, malaise, and eosinophilia. However, in our patient, the clinical profile was very nonspecific and therefore the diagnosis was primarily biochemical.

The diagnosis of this process is mainly made by exclusion since we do not have biomarkers that allow a definitive diagnosis; one of the most widely used scales at present is the Council for International of Medical Sciences/Reoussel Uclaf Causality Assessment Method (CIOMS/RUCAM) [4]. This scale tries to establish the causal relationship between the responsible drug and liver damage. It involves a scoring system (Table 2) [5] that categorizes the suspicion as “definite or very probable” (score >8), “probable” (score 6–8), “possible” (score 3–5), “unlikely” (score 1–2) and “excluded” (score ≤0). The case described presented a score of six points which, according to this scale, points to a probable diagnosis of LMWH-induced hepatotoxicity.

**Table 2:** CIOMS/RUCAM score for DILI assessment (modified with permission from Danan G., et al. [5]).

CIOMS/RUCAM						
Type of liver injury	Hepatocellular		Value	Cholestatic/mixed		Value
Chronological criteria	First exposure	Second exposure		First exposure	Second exposure	
Time of intake of medication at the beginning of the symptoms	5–90 days <5 or >90 days	1–15 days >15 days	2 1	5–90 days <5 or >90 days	1–90 days >90 days	2 1
Withdrawal time medication at the beginning of the symptoms	<15 days	≥15 days	1	≤30 days	≤30 days	1
**Course of the disease**	**Difference between maximum ALT value and limit normal upper**			**Difference between maximum ALP value and limit normal upper**		
Upon withdrawal of the medication	>50% improvement in eight days >50% improvement in 30 days Information is missing or there is no improvement Worsening or improvement <50% in 30 days		3 2 0 −1	>50% improvement in 180 days <50% improvement in 180 days Information is missing or there is no improvement		2 1 0
Risk factors	Age (≥55 years) Alcohol consumption		1 1	Age (≥55 years) Alcohol consumption or pregnancy		1 1
Concomitant therapy	None or unknown Drug with suggestive contribution Known hepatotoxin, suggestive contributions Proven role in case No information available		0 −1 −2 −3 0	None or unknown Drug with suggestive contribution Known hepatotoxin, suggestive contributions Proven role in case No information available		0 −1 −2 −3 0
Exclusion of other causes not medicated	Discarded Possible to not investigated Other probable cause		2 −2 to 1 −3	Discarded Possible to not investigated Other probable cause		2 −2 to 1 −3
Information of previous hepatotoxicity	Unknown reaction Posted but not labeled in drug-induced Labeled in the characteristics of the drug		2 From −2 to 1 −3	Unknown reaction Posted but not labeled in drug-induced Labeled in the characteristics of the drug		2 From −2 to 1 −3
Answer to re-administration of drug	Positive Compatible Negative Not available or not interpretable Plasma concentrations known as toxic		3 1 −2 0 3	Positive Compatible Negative Not available or not interpretable Plasma concentrations known as toxic		3 1 −2 0 3
Lab test validated with good predictive values	Positives Negatives Not available or not interpretable		3 −3 0	Positives Negatives Not available or not interpretable		3 −3 0

CIOMS/RUCAM, Council for International of Medical Sciences/Reoussel Uclaf Causality Assessment Method; ALP, alkaline phosphatase; ALT, alanine amino transferase.

On the other hand, there are a series of analytical/biochemical criteria for discontinuation of LMWH treatment due to the risk of hepatotoxicity. Most of them are based on an increase in AST values three times above the upper limit of the reference range, associated with another biochemical alteration such as an increase in bilirubin values or an alteration in the INR [10]. Isolated elevation of transaminases when this increase is prolonged for more than two weeks is also a criterion for discontinuation.

In the case of LMWH, the drug is often already discontinued at the time the diagnosis is made, as was our case. In those patients in whom an early diagnosis is achieved, it is recommended that treatment be replaced by fondaparinux, a synthetic and selective inhibitor of factor Xa, which acts by potentiating the effect of antithrombin III. It has been shown that the introduction of replacement therapy with fondaparinux does not affect the decrease in liver enzymes [4].

It is important to keep this cause of hepatotoxicity in mind in order to diagnose it early, using appropriate scales and avoiding subjecting the patient to unnecessary invasive diagnostic studies.

## Learning points

– Heparin-induced hepatitis is an uncommon and underdiagnosed adverse effect.
– The laboratory has a key role in early diagnosis as most patients do not present clinical manifestations at the onset.
– There are two patterns, hepatotoxic, present in most patients, and cholestatic, which is more infrequent.
– Withdrawal of the drug allows the patient to recover, usually after 14 days of suspension. In the cholestatic pattern, biochemical alterations take longer to normalize.
– Hepatitis occurs more frequently in patients treated with LMWH than in those using UFH, with fondaparinux being the main therapeutic alternative.
